# Global Safety Assessment of Adverse Events of Special Interest Following 2 Years of Use and 772 Million Administered Doses of mRNA-1273

**DOI:** 10.1093/ofid/ofae067

**Published:** 2024-02-02

**Authors:** Veronica Urdaneta, Daina B Esposito, Priyadarshani Dharia, Margot Stam Moraga, Kate Anteyi, Titi Oduyebo-Omotosho, Melissa Rossi, Paul Burton, José M Vega, Rachel Dawson, Walter Straus

**Affiliations:** Clinical Safety and Risk Management, Moderna, Inc, Cambridge, Massachusetts, USA; Medical Affairs, Moderna, Inc, Cambridge, Massachusetts, USA; Medical Affairs, Moderna, Inc, Cambridge, Massachusetts, USA; Clinical Safety and Risk Management, Moderna, Inc, Cambridge, Massachusetts, USA; Clinical Safety and Risk Management, Moderna, Inc, Cambridge, Massachusetts, USA; Clinical Safety and Risk Management, Moderna, Inc, Cambridge, Massachusetts, USA; Clinical Safety and Risk Management, Moderna, Inc, Cambridge, Massachusetts, USA; Medical Affairs, Moderna, Inc, Cambridge, Massachusetts, USA; Clinical Safety and Pharmacovigilance, Moderna, Inc, Cambridge, Massachusetts, USA; Clinical Safety and Pharmacovigilance, Moderna, Inc, Cambridge, Massachusetts, USA; Medical Affairs, Moderna, Inc, Cambridge, Massachusetts, USA; Clinical Safety and Pharmacovigilance, Moderna, Inc, Cambridge, Massachusetts, USA

**Keywords:** COVID-19, mRNA-1273, pharmacovigilance, SARS-CoV-2, SPIKEVAX

## Abstract

**Background:**

Large-scale use of mRNA COVID-19 vaccines during the pandemic was associated with enhanced safety monitoring to ensure accurate and timely review of safety. We reviewed the mRNA-1273 (original strain) safety profile following 2 years of use (>772 million administered doses), primarily focusing on predefined safety topics (ie, adverse events of special interest [AESIs]) proposed in advance of COVID-19 vaccine use.

**Methods:**

Cumulative mRNA-1273 safety data were included from spontaneous adverse event (AE) cases reported to Moderna’s global safety database between 18 December 2020 and 17 December 2022. Reporting rates of AESIs were calculated per 1 million doses of mRNA-1273 administered. Observed-to-expected (OE) ratios were computed by comparing observed rates of AESIs with the background/expected rate for these events to evaluate potential associations with mRNA-1273.

**Results:**

There were 658 759 identified case reports associated with 2 517 669 AEs. Most AEs were nonserious (83.4%; 2 098 954/2 517 669). Overall 0.7% (17 751/2 517 669) were fatal. AESIs represented 13.7% of all AEs (344 921/2 517 669), with reporting rates for most AESIs below the expected background incidence. Exceptions included anaphylaxis (OE ratio 3 days after vaccination, 2.09; 95% CI, 1.93–2.25) and, among individuals aged 12 to 40 years, myocarditis (OE ratio 7 days after any dose, 3.89 [3.50–4.32]; among men after dose 2, 8.57 [6.88–10.68]) and pericarditis (OE ratio 7 days after vaccination, 3.47; 2.89–4.16).

**Conclusions:**

This safety analysis of mRNA-1273 identified evidence of increased risk for anaphylaxis, myocarditis, and pericarditis but not for other AESIs identified for enhanced monitoring ahead of COVID-19 vaccine use.

## Teaser

This analysis evaluated observed-to-expected ratios for adverse events of special interest that were proposed in advance of COVID-19 vaccine use. Following administration of an estimated 772 908 958 doses of mRNA-1273, meaningful associations were found only for anaphylaxis, myocarditis, and pericarditis.

The introduction and authorization of COVID-19 vaccines had a major impact on decreasing COVID-19–associated hospitalizations and deaths during the pandemic [1–4]. Among these were 2 first-in-class mRNA vaccines: mRNA-1273 (SPIKEVAX; Moderna) and BNT162b2 (COMIRNATY; Pfizer and BioNTech Manufacturing GmbH). In a phase 3 randomized controlled trial of mRNA-1273, 2 doses were well tolerated and highly effective against symptomatic infection [5]. Real-world studies of mRNA-1273 subsequently demonstrated vaccine effectiveness against the ancestral SARS-CoV-2 strain and emergent variants of concern in the general population and among those at high risk of severe disease [6–11]. From initial authorization through December 2022, >248 million and >152 million doses have been administered in the United States and European Union, respectively [12].

Ensuring vaccine safety is essential to preserving public trust in vaccines, particularly during a pandemic and with a new vaccine platform [13, 14]. Newly introduced or expanded safety systems (eg, Biologics Effectiveness and Safety, Vaccine Safety, Vaccine Adverse Event Reporting System, Vaccine Safety Datalink, and Vaccine Monitoring Collaboration for Europe) have been implemented in the United States and the European Union to complement existing public health infrastructure and support reporting requirements for vaccine manufacturers [15, 16]. These postauthorization safety monitoring measures are designed to detect emergent and rare vaccine-related safety concerns not identified in clinical trials due to inclusion/exclusion criteria and limitations in sample size [13, 15, 17]. In May 2020, in anticipation of COVID-19 vaccine development, the Brighton Collaboration—in support of the Coalition for Epidemic Preparedness Innovations’ Safety Platform for Emergency Vaccines (SPEAC)—introduced a list of relevant adverse events of special interest (AESIs) to help guide clinical trial and postmarketing data analysis. These AESIs were based on immune-mediated COVID-19 sequelae or events common to many licensed vaccines with the potential to be observed in recipients of COVID-19 vaccines [18].

Following emergency use authorization of COVID-19 vaccines, data emerged suggesting an increased risk for several AESIs after vaccination [19–21]. In December 2020, reports surfaced of allergic reactions (anaphylaxis) following receipt of the mRNA COVID-19 vaccines [22]. In April 2021, there were reports of myocarditis and/or pericarditis (inflammation of the myocardium and pericardium, respectively) in individuals who had received one of the authorized mRNA COVID-19 vaccines [19]. At that time, the Advisory Committee for Immunization Practice presented data on cases of thrombosis with thrombocytopenia syndrome (TTS), a condition characterized by the formation of blood clots combined with low platelet count [23]. Several months later, the committee reviewed cases of Guillain-Barré syndrome (GBS; an autoimmune reaction primarily affecting peripheral nerves) following COVID-19 vaccination [24].

Herein, we review 2 years of safety data for mRNA-1273 from spontaneous adverse event (AE) case reports submitted to the Moderna global safety database (GSDB) according to previously defined AESIs, by which time >772 million doses of mRNA-1273 had been administered. This analysis includes estimation of reporting rates and observed-to-expected (OE) analysis, a quantitative pharmacovigilance approach used to estimate whether observed rates of certain AESIs after vaccination exceeded background rates [25].

## METHODS

### Data Sources

The Moderna GSDB collects safety data from spontaneous reports that are submitted by regulatory authorities, health care providers, and consumers where mRNA-1273 is authorized for use. Safety data obtained through sponsored clinical or observational studies, as well as cases extracted from secondary sources, such as events identified in the literature (MEDLINE, Embase), are added to the GSDB. The Medical Dictionary for Regulatory Activities (MedDRA), comprising internationally standardized terminology, was used to code events. This noninterventional retrospective database study used mostly AE reports submitted voluntarily to the Moderna GSDB, as required by regulatory authorities. A central institutional review board (Advarra) confirmed that this study met criteria for an exemption from oversight under 45 CFR 46.104(d)(4).

From 18 December 2020 (first international emergency use authorization issuance) to 17 December 2022, the GSDB was reviewed for valid case reports: those with an identifiable reporter, patient, adverse reaction, and suspected product. This search was conducted according to the list of AESIs (published May 2020 and updated October 2022) [26] and by using standard and/or customized queries (eg, standardized queries, high-level terms, preferred terms [PTs]) from the most updated version of the MedDRA (version 25.1) and excluding clinical trial data. The search strategies were based on well-defined approaches sourced from regulatory bodies (eg, SPEAC, Centers for Disease Control and Prevention, World Health Organization). AEs following immunization may include any untoward medical occurrence that follows administration and that does not necessarily have a causal relationship with vaccination [27]. This analysis was restricted to reports received for mRNA-1273 (original strain).

### Assessment of Case Reports

AESIs were continuously and systematically reviewed by Moderna as part of the standard process of identification and evaluation of possible safety risks associated with mRNA-1273. This process includes signal detection, validation, prioritization, evaluation, action recommendations, and documentation and tracking of signals; it follows the principles of the Good Pharmacovigilance Practices Module IX for Signal Management. Formal pharmacovigilance case definitions were used to categorize AEs/AESIs. A serious AE was defined as one that results in death, hospitalization, disability/permanent damage, or other events jeopardizing the patient that might require medical/surgical intervention [28]. Events of death were evaluated by safety medical personnel and included causality assessment based on the World Health Organization standardized causality assessment system [29]. Nonserious AEs were not considered for specific review.

Suspected cases of myocarditis and pericarditis were evaluated with the case definitions of the Centers for Disease Control and Prevention and the Brighton Collaboration [18, 30]. TTS-related PTs included cases with a thromboembolic event, which were then cross-checked for any of the following thrombocytopenia-related PTs: acquired amegakaryocytic thrombocytopenia, megakaryocytes decreased, platelet count decreased, platelet maturation arrest, platelet production decreased, platelet toxicity, thrombocytopenia, megakaryocytes abnormal, platelet count abnormal, platelet disorder, plateletcrit abnormal, plateletcrit decreased, immune thrombocytopenia, HELLP syndrome, thrombotic thrombocytopenic purpura, thrombocytopenic purpura, platelet transfusion, petechiae, ecchymosis, and purpura. GBS cases were identified via the following PTs: acute motor axonal neuropathy, acute motor sensory axonal neuropathy, Bickerstaff encephalitis, chronic inflammatory demyelinating polyradiculoneuropathy, demyelinating polyneuropathy, GBS, Miller Fisher syndrome, and subacute inflammatory demyelinating polyneuropathy.

### Statistical Analysis

AEs reported to the GSDB were reviewed and used for this analysis. Cases of AESIs were characterized with descriptive statistics according to age, sex, MedDRA PT, time to onset (TTO) after vaccination, and dose number. Reporting rates were used to estimate the frequency of specific AESIs, calculated as the number of cases per 1 million doses administered. In this analysis, the doses administered were conservatively estimated as 58% of doses distributed globally. Vaccine exposure in different age groups and by sex was estimated by the published distributions of COVID-19 vaccine recipient demographics in the United States per the Centers for Disease Control and Prevention as of 17 December 2022 [31].

### OE Analysis

OE ratios were determined for AESIs in this analysis, complementary to the continuous use of disproportionality. Expected incidence rates were identified from published sources [32–46], with an emphasis on recent large population-based studies of high quality. For estimation of observed reporting rates, all reports were included regardless of causality or TTO. An assumed risk window of 21 days was assigned after each administered vaccine dose to estimate exposed person-time at risk, unless otherwise specified. This window was selected for consistency with analyses that have been conducted by the US Vaccine Safety Datalink. The sum of all person-time was then used as a denominator to calculate a reporting rate comparable to expected rates from published sources. These expected rates were then multiplied by the estimated exposed person-time to identify the count of expected cases [25]. The observed reporting rate for each AESI was divided by the expected rate and presented alongside its associated 95% CI, calculated as follows: *e*^{log(IRR) ± 1.96 × SE[log(IRR)]}^ [25]. Age- and sex-stratified assessments of OE ratios were also performed. Subpopulation evaluations presented in this analysis included children (<18 years), adults (18–64 years), and elderly individuals (≥65 years). For anaphylaxis, a 3-day risk window was applied to person-time and case counts; a 7-day risk window, stratified by dose, was used for myocarditis and pericarditis.

For any AESI where the lower bound of the 95% CI was >1 in overall or subgroup analyses, cases were medically reviewed to determine the plausibility of an association. In some instances, expected background incidence rates could not reasonably be determined because of sparse or absent epidemiologic data in the general population, resulting in more qualitative review of case characteristics.

This OE analysis reports on all AESIs initially proposed by SPEAC for COVID-19 vaccines and provides additional information on those that received attention by the Advisory Committee for Immunization Practice during the first 6 months following emergency use: anaphylaxis, myocarditis, pericarditis, TTS, and GBS.

Additional details of the statistical methods used in these analyses are provided in the supplementary materials.

## RESULTS

### Cases Identified

As of 17 December 2022, 1 315 589 716 doses of mRNA-1273 have been distributed to 91 countries, with ∼772 908 958 doses administered. During this period, 658 759 cases were captured in the mRNA-1273 GSDB, which included 2 517 669 AEs (AESIs represented 13.7% of AEs; Table 1). The overall reporting rate was 852.3 cases per 1 million doses administered.

**Table 1.** Characteristics of Cases Reported to the Moderna Global Safety Database From December 2020 to December 2022

	All		Children (<18 y)		Adults (18 to <65 y)		Elderly (≥65 y)	
	No.	%	No.	%	No.	%	No.	%
All	658 759	100.0	10 036	1.5	463 847	70.4	133 331	20.2
AE case reporting rate per 10 000 doses administered	8.52		4.33		8.34		6.90	
Age, y								
<2	494	0.1	494	4.9				
2–4	421	0.1	421	4.2				
5–11	569	0.1	569	5.7				
12–17	8552	1.3	8552	85.2				
18–24	37 085	5.6			37 085	8.0		
25–39	158 961	24.1			158 961	34.3		
40–49	114 149	17.3			114 149	24.6		
50–64	153 652	23.3			153 652	33.1		
65–74	81 159	12.3					81 159	60.9
≥75	52 174	7.9					52 174	39.1
Missing	51 546	7.8						
Sex								
Male	186 791	28.4	4302	42.9	128 723	27.8	43 607	32.7
Female	444 791	67.5	5196	51.8	329 120	71.0	87 528	65.6
Missing	27 177	4.1	538	5.4	6004	1.3	2196	1.6
Region								
Europe	338 208	51.3	2435	30.1	279 372	60.2	40 295	30.2
North America	263 069	39.9	5150	58.5	141 849	30.6	85 191	63.9
Asia	47 150	7.2	1152	11.5	35 413	7.6	6801	5.1
Oceania	7635	1.2	570	1.9	5671	1.2	771	0.6
Latin America/Caribbean	2486	0.4	706	7.0	1433	0.3	271	0.2
Middle East	183	0.0	23	0.2	99	0.0	5	0.0
Africa	34	0.0	0	0.0	9	0.0	0	0.0
Unknown	2	0.0	0	0.0	0	0.0	0	0.0
Comorbidity in the case report								
None	382 132	58.0	8130	75.4	276 842	59.7	58 844	44.1
At least 1	276 627	42.0	1906	24.6	187 005	40.3	74 487	55.9
5 most frequent in medical history before vaccination^a^								
Drug hypersensitivity	55 517	8.4	139	1.4	35 458	7.6	19 420	14.6
Hypertension	34 350	5.2	17	0.0	15 705	3.4	18 201	13.7
COVID-19	20 013	3.0	101	1.0	15 478	3.3	3542	2.7
Asthma	17 469	2.7	174	1.7	13 416	2.9	3522	2.6
Food allergy	15 057	2.3	76	0.8	11 280	2.4	3524	2.6

Abbreviation: AE, adverse event.

^a^The 5 most common medical history terms varied by age and included maternal exposure during breastfeeding (n = 96, 0.96%) for children and diabetes mellitus (n = 5689, 4.3%) and hypothyroidism (n = 3672, 2.8%) for elderly adults.

**Figure 1.**
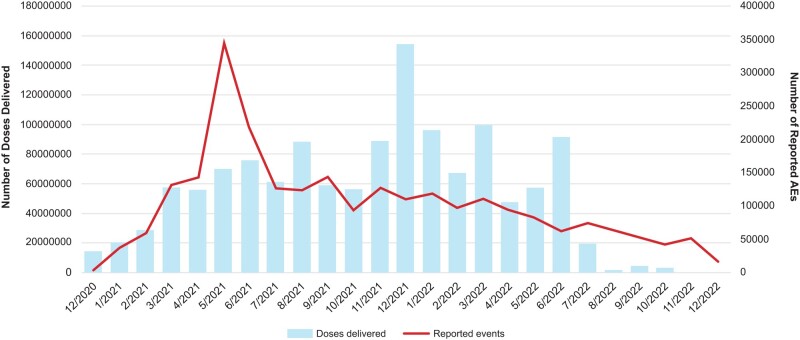
mRNA-1273 doses distributed and reported adverse events (AEs) by month from December 2020 to December 2022.

In the first 6 months of vaccine distribution, the number of events increased as the number of doses distributed increased, with peak events reaching 344 842 in May 2021 (Figure 1). Since then, events gradually declined, with 16 706 events in December 2022.

Most cases were reported in individuals aged 18 to 64 years (70.4%) and women (67.5%). While the majority of cases were from Europe (338 208 cases, 51.3%) and North America (263 069 cases, 39.9%), cases were also from Asia (47 150 cases, 7.2%), Oceania (7635 cases, 1.2%), Latin America/Caribbean (2486 cases, 0.4%), the Middle East (183 cases, 0%), and Africa (34 cases, 0%). In 42% of cases, ≥1 underlying health condition was documented before vaccination; the most common conditions across age groups were drug hypersensitivity (8.4%), hypertension (5.2%), COVID-19 (3.0%), asthma (2.7%), and food allergy (2.3%; Table 1).

**Figure 2.**
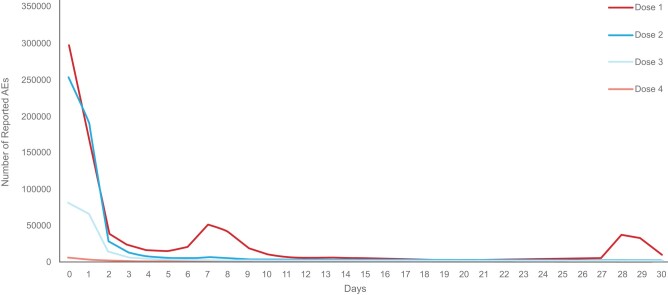
Time to onset of reported adverse events (AEs) with mRNA-1273 by dose.

Most AEs were nonserious (83.4%) and reported by regulatory health authorities (79.7%). The most frequent AEs in the overall population are summarized in Table 2. Of the AEs, 49.0% were associated with reactogenicity, which included local events (eg, injection site pain, redness/swelling at the injection site) and systemic events (eg, myalgia, arthralgia, fever, headache). A higher number of AEs occurred after dose 1 (30.1%) as compared with subsequent doses. The median TTO from the most recent dose was 3 days (Figure 2). At the time of receipt, the outcome was recovered/recovering for 47.4% of AEs. Less than 1% of all AEs (0.7%) were fatal; this varied by age, with 70.4% of fatal AEs occurring in elderly adults. No cause of death was reported for 16.9% of all fatal events. For those cases reporting cause of death, the leading event was COVID-19 (3.9% of all fatal events), followed by dyspnea (3.0%) and cardiac arrest (2.6%).

**Table 2.** Characteristics of Adverse Events Reported to the Moderna Global Safety Database From December 2020 to December 2022

	All		Children (<18 y)		Adults (18 to <65 y)		Elderly (≥65 y)	
	No.	%	No.	%	No.	%	No.	%
All	2 517 669	100.0	21 508	0.9	1 890 311	75.1	476 590	18.9
Event seriousness								
Serious^a^	418 715	16.6	2875	13.4	291 077	15.4	101 808	21.4
Nonserious	2 098 954	83.4	18 633	86.6	1 599 232	84.6	374 784	78.6
HCP submitted the report	1 074 760	42.7	15 479	72.0	755 525	40.0	269 105	56.5
Reporter type								
Regulatory authority	2 007 048	79.7	16 385	76.2	1 623 913	85.9	312 856	65.6
Spontaneous report^b^	502 516	20.0	5004	23.3	260 874	13.8	162 579	34.1
Other (eg, literature)	8105	0.3	119	0.6	5522	0.3	1157	0.2
Dose number								
1	758 268	30.1	8692	40.4	563 405	29.8	162 958	34.2
2	606 530	24.1	3529	16.4	470 410	24.9	121 408	25.5
3	202 370	8.0	405	1.9	164 262	8.7	28 139	5.9
4	11 467	0.5	53	0.2	4251	0.2	6512	1.4
Other/not reported	939 034	37.3	8829	41.0	687 983	36.4	157 575	33.1
Event outcome at the time of report								
Fatal	17 751	0.7	60	0.3	4723	0.2	12 501	2.6
Recovered/recovering	1 192 627	47.4	11 539	53.6	944 247	50.0	191 273	40.1
Not recovered	709 987	28.2	4208	19.6	562 760	29.8	120 000	25.2
Not reported	597 304	23.7	5701	26.5	378 581	20.0	152 816	32.1
Most frequently reported events								
Headache	146 039	5.8	933	4.3	119 374	6.3	19 468	4.1
Pyrexia	139 460	5.5	1435	6.7	111 181	5.9	19 874	4.2
Fatigue	127 184	5.1	452	2.1	102 437	5.4	19 198	4.0
Chills	97 683	3.9	243	1.1	77 877	4.1	15 664	3.3
Myalgia	91 510	3.6	339	1.6	75 322	4.0	11 849	2.5
Injection site pain	70 431	2.8	118	0.6	61 471	3.3	7463	1.6
Nausea	69 913	2.8	377	1.8	55 846	3.0	10 834	2.3
Malaise	67 283	2.7	266	1.2	56 903	3.0	8033	1.7
Arthralgia	55 998	2.2	161	0.8	44 327	2.3	9290	2.0
Pain in extremity	53 606	2.1	319	1.5	37 563	2.0	12 261	2.6

Abbreviation: HCP, health care provider.

^a^A serious adverse event was defined as one that results in death, hospitalization, disability/permanent damage, or other events jeopardizing the patient that might require medical/surgical intervention.

^b^For spontaneous case reports, event outcomes were those provided at the time of adverse event report submission and did not often contain important elements of the adverse event, including any follow-up information.

### Adverse Events of Special Interest

#### Anaphylaxis

Based on cumulative exposure and published background rates, ∼6577 cases of anaphylaxis were expected <21 days postvaccination. There were 2588 cases (2651 events) of anaphylaxis (0.4% of all cases); most were in women (73.6%). Of these cases, 67 (2.6%) included a fatal outcome; however, most cases (98.5%) were unrelated to vaccination. When examined by dose number, 47.0% of cases occurred after dose 1, 19.7% after dose 2, 8.3% after dose 3, and 0.8% after dose 4; 24.2% did not indicate the dose number. Most events occurred on the same day of vaccination (63.3%) regardless of the dose number.

The reporting rate of anaphylaxis was 3.35 cases per 1 million doses administered, with a higher rate in adults (18–64 years old, 3.79 cases per 1 million doses; Table 3). Considering a standard 21-day interval assessed across AESIs, the reporting rate was below published population-based rates (OE ratio, 0.39; 95% CI, .38–.41). However, when comparisons involved events within a 3-day risk window, which is a more plausible time frame for this AE, there was an overall increase above background rates (OE ratio, 2.09; 95% CI, 1.93–2.25), among women (3.13; 95% CI, 2.82–3.47), and in adults aged 18 to 64 years (4.01; 95% CI, 3.60–4.46). Consideration of background rates from alternate published studies produced consistent conclusions [45].

**Table 3.** Reporting Rates of AESI by Age and Sex From December 2020 to December 2022

	Reporting Rate Per 1 Million Doses Administered							
		Sex		Age, y				
AESI	All	Male	Female	<18	18 to <65	≥65	Total Cases Reported, %	OE Ratio (95% CI)^a^
Acute respiratory distress	0.79	0.91	0.67	0.00	0.31	2.28	0.093	0.06 (.05–.06)
Multisystem inflammatory syndrome	0.21	0.19	0.23	0.22	0.16	0.32	0.025	0.23 (.2–.27)
Myocarditis with or without pericarditis								
21-d risk window	5.68	8.63	2.68	8.37	6.13	1.32	0.666	1.1 (1.05–1.15)
7-d risk window	2.88	4.77	1.09	5.69	3.54	0.37	0.337	1.67 (1.56–1.78)
Age 12–40 y	7.46	6.36	1.06				0.258	3.89 (3.5–4.32)
Dose 1, age 12–40 y	2.55						0.042	1.33 (1.11–1.59)
Male		1.96					0.000	1.6 (1.29–1.99)
Female			0.51				0.000	0.84 (.6–1.18)
Dose 2, age 12–40 y	11.81						0.131	6.16 (5.16–7.37)
Male		7.01					0.000	8.57 (6.88–10.68)
Female			0.75				0.000	1.84 (1.29–2.61)
Dose 3, age 12–40 y	4.69						0.031	2.45 (1.89–3.16)
Male		1.49					0.000	3.07 (2.25–4.18)
Female			0.34				0.000	1.39 (.86–2.24)
Pericarditis without myocarditis								
21-d risk window	2.89	3.23	2.52	1.77	3.16	1.40	0.339	0.93 (.88–.99)
7-d risk window	1.25	1.44	1.06	0.99	1.49	0.46	0.146	1.21 (1.1–1.32)
Age 12–40 y	2.26	2.85	1.70				0.078	3.47 (2.89–4.16)
Dose 1, age 12–40 y	1.07						0.018	1.64 (1.22–2.2)
Male		0.61					0.000	0.99 (.71–1.39)
Female			0.42				0.000	1.12 (.75–1.67)
Dose 2, age 12–40 y	2.26						0.025	3.47 (2.52–4.79)
Male		1.03					0.000	2.51 (1.78–3.55)
Female			0.43				0.000	1.71 (1.09–2.69)
Dose 3, age 12–40 y	1.27						0.008	1.95 (1.24–3.07)
Male		0.34					0.000	1.39 (.84–2.28)
Female			0.15				0.000	1.02 (.53–1.95)
Other forms of cardiac injury								
Arrhythmia	11.39	9.71	12.61	1.51	10.62	13.09	1.336	0.03 (.03–.03)
Heart failure	1.78	2.04	1.50	0.00	0.96	4.20	0.209	0.02 (.02–.02)
Ischemic coronary artery disease	2.33	3.01	1.62	0.04	1.67	4.04	0.274	0.01 (.01–.01)
Microangiopathy	0.04	0.05	0.03	0.00	0.03	0.05	0.004	0.1 (.07–.15)
Stress cardiomyopathy	0.08	0.02	0.12	0.00	0.03	0.19	0.009	0.02 (.01–.02)
Any thromboembolic event	16.41	16.52	15.77	0.65	12.35	27.24	1.926	0.14 (.14–.14)
Hemorrhagic disorders including DIC								
Deep vein thrombosis	6.67	6.79	6.44	0.39	5.57	9.97	0.783	0.02 (.02–.03)
Disseminated intravascular coagulation	0.05	0.04	0.06	0.00	0.04	0.08	0.006	0.01 (.01–.01)
Cerebral venous sinus thrombosis	0.26	0.25	0.27	0.04	0.28	0.21	0.031	0.2 (.18–.24)
Pulmonary embolism	4.09	4.12	3.99	0.17	3.29	6.44	0.480	0.05 (.04–.05)
Splanchnic venous thrombosis	0.09	0.11	0.07	0.00	0.08	0.12	0.010	0.06 (.05–.08)
Stroke								
All	5.84	5.91	5.60	0.39	3.53	12.17	0.685	0.04 (.04–.04)
Hemorrhagic	0.96	0.97	0.92	0.17	0.61	1.96	0.113	0.01 (.01–.02)
Anosmia/ageusia	4.22	2.68	5.38	0.60	4.10	3.84	0.495	0.55 (.53–.58)
Chilblain-like lesions	0.14	0.07	0.19	0.04	0.13	0.10	0.016	0.02 (.01–.02)
Erythema multiforme^b^	0.44	0.34	0.50	0.69	0.48	0.22	0.051	0.12 (.11–.13)
Single organ cutaneous vasculitis	0.47	0.30	0.61	0.13	0.42	0.61	0.056	0.17 (.15–.19)
Acute								
Kidney injury	1.39	1.55	1.23	0.17	0.67	3.44	0.163	0.01 (.01–.01)
Liver injury	0.49	0.39	0.56	0.13	0.42	0.65	0.058	0.15 (.13–.16)
Pancreatitis	0.36	0.35	0.36	0.00	0.32	0.47	0.042	0.17 (.15–.19)
Rhabdomyolysis	0.22	0.23	0.20	0.00	0.16	0.37	0.026	0.14 (.12–.16)
Subacute thyroiditis^c^	0.58	0.23	0.90	0.09	0.70	0.20	0.068	0.03 (.02–.03)
Anaphylaxis								
21-d risk window	3.35	1.68	4.74	0.95	3.79	1.74	0.393	0.39 (.38–.41)
3-d risk window	2.53	1.20	3.65	0.69	3.03	1.10	0.297	2.09 (1.93–2.25)
Thrombocytopenia	3.05	2.30	3.61	1.21	2.57	4.11	0.358	0.09 (.09–.1)
Generalized convulsion	4.39	3.87	4.62	3.41	4.71	2.89	0.515	0.22 (.21–.22)
Acute disseminated encephalomyelitis	0.14	0.14	0.13	0.13	0.14	0.11	0.016	0.43 (.34–.53)
Guillain-Barré syndrome	0.88	0.94	0.79	0.22	0.77	1.13	0.104	0.38 (.35–.42)
Acute aseptic arthritis	3.84	2.14	5.24	0.13	3.47	4.35	0.450	0.07 (.06–.07)
Aseptic meningitis	0.21	0.19	0.22	0.04	0.22	0.14	0.024	0.05 (.04–.06)
Encephalitis/encephalomyelitis								
Encephalitis	0.36	0.35	0.36	0.17	0.34	0.38	0.042	0.35 (.3–.4)
Multiple sclerosis	0.48	0.22	0.71	0.04	0.54	0.23	0.056	0.05 (.04–.05)
Myasthenia gravis	0.22	0.26	0.17	0.04	0.12	0.48	0.026	0.51 (.42–.61)
Optic neuritis	0.18	0.13	0.22	0.13	0.20	0.09	0.021	0.06 (.05–.07)
Fibromyalgia	0.42	0.06	0.72	0.00	0.41	0.39	0.049	0.01 (.01–.01)
Transverse myelitis	0.16	0.15	0.17	0.00	0.13	0.22	0.019	0.22 (.18–.27)
Bell palsy	4.37	3.81	4.76	0.82	4.33	4.30	0.513	0.12 (.12–.13)
Thrombocytopenia and thrombosis	0.30	0.32	0.27	0.09	0.20	0.55	0.035	0.32 (.28–.38)
Immune thrombocytopenia	0.46	0.37	0.53	0.35	0.38	0.61	0.054	0.12 (.11–.13)
CLS^d^	0.03	0.02	0.04	0.00	0.01	0.08	0.004	0.54 (.33–.89)
Delayed hypersensitivity reaction^e^	0.37	0.25	0.49	0.04	0.40	0.34	0.044	Not estimable
Extensive limb swelling^**e**^	4.84	1.18	8.07	0.23	6.03	1.83	0.568	Not estimable
Facial swelling (with dermal fillers)^e^	4.67	1.65	7.20	0.58	3.78	3.77	0.548	Not estimable
Dizziness^e^	60.53	22.99	83.53	11.96	68.33	37.12	7.102	Not estimable
Tinnitus	8.49	6.80	9.66	0.66	9.49	4.99	0.996	0.27 (.27–.28)

Abbreviations: AESI, adverse event of special interest; CLS, capillary leak syndrome; DIC, disseminated intravascular coagulation; OE, observed to expected.

^a^OE analyses were conducted with a 21-day risk window unless otherwise specified.

^b^Includes target lesions and toxic skin eruption.

^c^Given limited feasibility of capturing subacute conditions in spontaneous adverse event reporting data, this outcome was defined as thyrotoxicosis.

^d^Systemic CLS is a rare disorder with a poorly characterized incidence rate. Fewer than 500 cases of CLS have been reported worldwide, and the prevalence of CLS has been reported as <1/1 000 000 [35, 47]. As such, validity of OE estimates is limited.

^e^For some outcomes for which population-based data characterizing a suitable expected event rate were not available, OE analyses could not be performed.

#### Myocarditis and/or Pericarditis

For myocarditis, 3999 cases were expected <21 days postvaccination, and 1333 were expected <7 days postvaccination. For pericarditis, 2400 and 800 cases were expected <21 and <7 days postvaccination, respectively. There were 6702 cases (7076 events) of myocarditis and/or pericarditis reported to the GSDB (0.7% and 0.5% of all cases). Most cases were in men aged 18 to 39 years (39.2%). Cases of myocarditis and pericarditis were most frequent after dose 2 (28.4%), and most had an onset <7 days after vaccination (65.5%). Most cases reported an outcome of recovered/recovering (46.9%), with 82 cases involving a fatal outcome. Of those fatal cases, 5 included events of myopericarditis, 7 pericarditis, 66 myocarditis, 1 giant cell myocarditis, and 4 carditis. Most fatal cases occurred in men (66.3%), with a median age of 58 years and a median TTO of 5 days after any dose. Of the fatal cases, most had an associated medical history that may have contributed to the fatal outcome (eg, cardiovascular disease, COVID-19 infection).

The overall reporting rates for myocarditis and pericarditis were 5.68 and 2.89 cases per 1 million doses administered, respectively (Table 3). The rate was higher in younger adults, as previously described [48]. For the overall population, the reporting rate of myocarditis was elevated but similar to the expected rate estimated in a US population-based study (OE ratio, 1.10; 95% CI, 1.05–1.15) for an assumed 21-day risk window. Myocarditis occurred at an increased rate <7 days postvaccination (OE ratio, 1.67; 95% CI, 1.56–1.78), especially among individuals aged 12 to 40 years (3.89; 95% CI, 3.50–4.32) and after dose 2 among men aged 12 to 40 years (8.57; 95% CI, 6.88–10.68). The rate of pericarditis was also elevated in individuals aged 12 to 40 years <7 days postvaccination (3.47; 95% CI, 2.89–4.16).

#### Thrombocytopenia Syndrome

For TTS, 711 cases were expected <21 days postvaccination. There were 230 cases (250 events) identified with TTS-related PTs (0.02% of all cases); of those, 31 had a fatal outcome. No major differences were observed in cases of men (50.9%) vs women (46.5%); the mean age was 59.5 years. When dose numbers and TTO were reported, cases most frequently occurred after dose 2 (29.2%) and >7 days postvaccination (n = 96, 38.4%). When the reporting rate was compared with population-based estimates from the ACCESS project [45], the most conservative estimate of the OE ratio was 0.32 (95% CI, .28–.38) based on an expected incidence (Table 3). No subgroup analyses for age or sex demonstrated an elevated reporting rate.

#### Guillain-Barré Syndrome

For GBS, 1778 cases were expected <21 days postvaccination. There were 683 cases (713 events) under GBS-related PTs (0.1% of total cases); of these, 673 were considered serious and 10 had fatal outcomes. There were more cases in men (51.1%) than women (46.7%), and most cases were in individuals aged >50 years. When dose numbers and TTO were reported, more cases occurred after dose 1 (24.3%) or dose 2 (23.7%) and >14 days after any dose (36.6%). The reporting rate for GBS was below a US population-based estimate of incidence (OE ratio, 0.38; 95% CI, .35–.42; Table 3). No subgroup analyses for age or sex demonstrated an elevated reporting rate.

#### Other AESIs

Among other AESIs, reporting rates varied across subgroups defined by age and sex. Overall and in all subgroups considered, the estimated reporting rates for these conditions were below or compatible with the expected background incidence rate, and none met the prespecified threshold of a 95% CI lower bound >1 in this analysis. Medical review of AESIs did not suggest any additional risks associated with the administration of mRNA-1273 (Table 3).

## DISCUSSION

The COVID-19 pandemic has placed extraordinary demands on organizations charged with the public health response. By defining AESIs in advance of authorization of COVID-19 vaccine use, SPEAC and the Brighton Collaboration provided a helpful framework to facilitate evaluation of AEs following immunization and AESIs that may occur in association with COVID-19 vaccines, whether incidentally or causally. To provide an update on the safety profile of mRNA-1273, we evaluated cumulative global AESIs entered into the Moderna GSDB from 18 December 2020 to 17 December 2022.

Pharmacovigilance efforts made during the COVID-19 pandemic have utilized background rates for conditions anticipated to be AEs, allowing for a more intuitive approach to characterizing strength of association and one more readily suited for additional analyses (eg, estimating strength of association categorized by age and sex). The quantitative evaluation of the strength of association for spontaneous reported data has often employed disproportionality testing, a form of data mining that evaluates the strength of association of individual AEs observed following immunization as compared with those observed following immunization with other vaccines [49]. In this analysis, OE ratios for most AESIs evaluated did not exceed the threshold of the lower bound of the 95% CI >1 for the standard risk window of 21 days following vaccination. Reports of certain AESIs occurred at a relatively low rate, with 2588 cases of anaphylaxis, 6702 of myocarditis and/or pericarditis, 230 of TTS, and 683 of GBS. However, as previously reported [48, 50, 51], AESIs of myocarditis with or without pericarditis and pericarditis demonstrated elevated reporting rates in subgroup analyses, with more marked associations observed in sensitivity analyses that restricted timing to a 7-day risk window after vaccination. Rates of anaphylaxis were also increased during the 3 days after vaccination. Our analysis confirmed an elevated risk for anaphylaxis, myocarditis, and/or pericarditis but not for TTS or GBS. Upon confirmation of these risks, anaphylaxis, myocarditis, and pericarditis were added to mRNA-1273 prescribing information as part of good pharmacovigilance practices and to keep health care professionals and the general public informed.

Given the large-scale use during the first 2 years after mRNA-1273 authorization (following the administration of >772 million doses), the percentage of AEs that were fatal following administration of mRNA-1273 was small (0.7%).

Information presented here offers a safety evaluation of a subset of cases submitted to the GSDB. However, an inherent limitation is the spontaneous nature of cases received through the international voluntary reporting system. Moreover, when observed reporting rates of AESIs and expected background rates are compared, there is a lack of visibility regarding patient-level data and limited availability of details on exposure in special risk groups who have received mRNA-1273. Additionally, published information on overall estimates of vaccine use varies, making it difficult to accurately estimate exposure to mRNA-1273, especially within population subgroups. Furthermore, it should be noted that many AESIs have highly variable estimates of incidence across sources. Confounding almost certainly contributes to the variability, as the proportion of mRNA-1273 vaccine recipients with relevant comorbidities and other risk factors for the outcomes assessed is unknown and subgroup analyses of potential interest are infeasible. Some of these limitations may be mitigated by confirmation via observational studies evaluating specific safety questions. Nonetheless, postmarketing surveillance allows for the collection of information on a large number of individuals across a range of medical practices, provides data regarding unstudied uses or populations, and can detect rare and unexpected AEs [52]. There has been a notable reduction in cases submitted to the GSDB following the peak in May 2021, which likely reflects an increasing familiarity with the safety profile of mRNA-1273, as well as the declining use of mRNA-1273 with the development and use of variant-containing vaccines.

## CONCLUSIONS

Among the list of AESIs proposed by SPEAC in advance of COVID-19 vaccine use and with the exceptions of anaphylaxis, myocarditis, and pericarditis, this mRNA-1273 safety analysis found no evidence to suggest an increased risk of AESI. Safety monitoring of mRNA-1273 and variant-containing vaccines is ongoing.

## Supplementary Material

ofae067_Supplementary_Data

## Supplementary Data

Supplementary materials are available at *Open Forum Infectious Diseases* online. Consisting of data provided by the authors to benefit the reader, the posted materials are not copyedited and are the sole responsibility of the authors, so questions or comments should be addressed to the corresponding author.

## Notes

***Acknowledgments.*** We thank Oketoun Akangbe, Vasudev Bhupathi, Magalie Emilebacker, Kevin McNamara, Janaki Ram Neelakanti, and the rest of the pharmacovigilance scientists who supported the highlighted topics, along with Andrea Sutherland, Vaishali Khamamkar, and Samantha St Laurent, for their contributions to the manuscript. Medical writing and editorial assistance were provided by Jared Mackenzie, PhD, Ashlea Inan, PhD, and Lindsey Kirkland, PhD, of MEDiSTRAVA in accordance with Good Publication Practice guidelines (GPP 2022), funded by Moderna, Inc, and under the direction of the authors.

***Author contributions.*** V. U., D. B. E., P. D., M. S. M., K. A., and W. S. conceived and designed the current study. V. U., D. B. E., P. D., and M. S. M. were involved with data collection. V. U., D. B. E., P. D., M. S. M., K. A., T. O.-O., M. R., and W. S. carried out analysis and interpretation of data and take responsibility for the integrity of the data and accuracy of the data analysis. All authors critically reviewed and revised the manuscript for important intellectual content and approved the final draft.

***Patient consent statement.*** This noninterventional retrospective database study used mostly adverse reports submitted voluntarily to the Moderna GSDB, as required by regulatory authorities. A central institutional review board (Advarra) confirmed that this study met criteria for an exemption from oversight under 45 CFR 46.104(d)(4) and is therefore exempt from patient informed consent requirements.

***Financial support*.** This work was supported by Moderna, Inc.
