# Supplementary material for: Global Safety Assessment of Adverse Events of Special Interest Following 2 Years of Use and 772 Million Administered Doses of mRNA-1273
Source: Open Forum Infect Dis. 2024 Feb 2;11(3):ofae067. doi: 10.1093/ofid/ofae067 (PMC10946654; doi:10.1093/ofid/ofae067)
Supplement: ofae067_Supplementary_Data [file ofae067_supplementary_data.docx]

**Supplementary Material**

**Supplementary Methods. Observed-to-expected ratios**

**Supplementary Methods. Observed-to-expected ratios**

Moderna has conducted observed/expected analyses using disease incidence rates identified in published sources with a focus on studies describing population-based rates of disease in the US prior to COVID-19. To calculate observed reporting rates, person-time at risk was estimated according to the following. A risk window of 21 days was assigned after each administered vaccine dose unless otherwise specified (eg, for myocarditis, analyses using a 7-day risk window were also performed). All mRNA-1273 (COVID-19 Vaccine, SPIKEVAX; Moderna Inc, Cambridge, MA, USA) doses were included in the estimate of global administered doses. This window was selected for consistency with analyses that have been conducted by the US Vaccine Safety Datalink. The sum of all person-time was then used as a denominator to calculate the reporting rate. Expected rates from published sources were then multiplied by the same person-time estimate to identify the count of expected cases.

Age, sex, and age by sex stratified assessments of observed to expected rates were additionally performed. Our knowledge of demographics for administration data is limited to the information tracked and published by health officials within the countries receiving the vaccine. Not all health authorities provided the same age strata when sharing this information, and these are not always aligned with age categories presented in published sources of external data on the estimated incidence of conditions of interest. Given the volume of mRNA-1273 doses administered in the US, we applied the US age distribution to the total administered doses of vaccine administered and corresponding person-time accrued. Because BNT162b2 (COVID-19 vaccine, COMIRNATY; Pfizer Inc, New York, NY, USA; BioNTech Manufacturing GmbH, Mainz, Germany) was authorized for use in adolescents (12-17 years) earlier than mRNA-1273 in the US, it is expected that most of the primary series COVID-19 vaccine doses seen in this age group are not mRNA-1273. To account for this, we limited the total assumed accrued exposure of the primary series in individuals aged <18 years to 3% of the total. The estimate of 3% was selected based on the assumption that adolescent use of mRNA-1273 for primary series vaccination in the US, where approximately one third of global administrations have occurred, was limited because authorization was restricted to individuals aged ≥18 years during the time that most people received a primary series. The doses in individuals aged <18 years were further distributed to reflect the age distribution as reported by Centers for Disease Control, which now includes more granular pediatric age bands. The age distribution of the first and second booster reflected the age distribution as reported by Centers for Disease Control without modification.

Additionally, sensitivity analyses were conducted to assess the potential impact of under reporting and/or data lags in reporting. In these assessments, observed to expected rate ratios were recalculated assuming that cases captured in the observed reporting rate represented 50% or 25% of the true exposed cases. These analyses were performed for all AESIs. Age and sex-specific incidence rates identified from published literature were then used to estimate expected cases within each category. Where age groups used in sources of background incidence did not align with the available grouping for population-based vaccine administration, the lowest logical adjacent category was used, considering the extent of age overlap and prevailing trends in disease incidence. Where sources did not include all required subgroups, available information was used to estimate missing categories. For example, for references including data for age and sex separately but not combined, age-specific incidence estimates within subgroups of sex were estimated by multiplying the applicable age-specific estimate by the ratio of the sex specific estimate to the overall estimate. Where no information on age- or sex-specific rates could be identified, overall rates were applied to all categories. Limitations in comparing observed reporting rates and expected background rates include lack of direct visibility regarding patient-level data for administration, and limited availability of details on exposure in special risk groups who have received mRNA-1273 booster vaccines (other than an expectation that governments will distribute mRNA-1273 booster vaccines to the indicated adult population). Further, it should be noted that many AESI have highly variable estimates of incidence across sources. As the observed reported cases are captured in a continuously updated surveillance database, cases may be revised or deleted month to month with accrual of significant information after initial receipt (eg, clarification of nature of the event, corrections to patient demographics, medical history, laboratory data, or procedures). For this reason, reduced case counts in age by sex stratified subgroups is occasionally observed when comparing counts from month to month. Because the proportion of mRNA booster vaccine recipients with relevant comorbidities and other risk factors for the outcomes assessed is unknown, it is possible that estimates are confounded, and subgroup analyses of potential interest are infeasible.
